# Interaction between *Plasmodium* Glycosylphosphatidylinositol and the Host Protein Moesin Has No Implication in Malaria Pathology

**DOI:** 10.3389/fcimb.2017.00183

**Published:** 2017-05-16

**Authors:** Josefine Dunst, Nahid Azzouz, Xinyu Liu, Sachiko Tsukita, Peter H. Seeberger, Faustin Kamena

**Affiliations:** ^1^Institute of Chemistry and Biochemistry, Free University BerlinBerlin, Germany; ^2^Parasitology Unit, Max Planck Institute for Infection BiologyBerlin, Germany; ^3^Department of Biomolecular Systems, Max Planck Institute for Colloids and InterfacesPotsdam, Germany; ^4^Department of Chemistry, University of PittsburghPittsburgh, PA, USA; ^5^Department of Frontier Biosciences, Graduate School of Frontier Biosciences, Osaka UniversityOsaka, Japan

**Keywords:** GPI, ERM, moesin, *Plasmodium*, cerebral malaria

## Abstract

Glycosylphosphatidylinositol (GPI) anchor of *Plasmodium falciparum* origin is considered an important toxin leading to severe malaria pathology through stimulation of pro-inflammatory responses from innate immune cells. Even though the GPI-induced immune response is widely described to be mediated by pattern recognition receptors such as TLR2 and TLR4, previous studies have revealed that these two receptors are dispensable for the development of severe malaria pathology. Therefore, this study aimed at the identification of potential alternative *Plasmodium* GPI receptors. Herein, we have identified the host protein moesin as an interaction partner of *Plasmodium* GPI *in vitro*. Given previous reports indicating the relevance of moesin especially in the LPS-mediated induction of pro-inflammatory responses, we have conducted a series of *in vitro* and *in vivo* experiments to address the physiological relevance of the moesin-*Plasmodium* GPI interaction in the context of malaria pathology. We report here that although moesin and *Plasmodium* GPI interact *in vitro*, moesin is not critically involved in processes leading to *Plasmodium*-induced pro-inflammatory immune responses or malaria-associated cerebral pathology.

## Introduction

Malaria still causes a devastatingly high number of deaths and new infections each year and is thereby a major contributor to the global burden of infectious diseases (WHO, 2016). This disease is caused by human host-adapted *Plasmodium* species and transmitted by the bite of an infective *Anopheles* mosquito. During *Plasmodium* infection, an immune response is mounted by the host in order to limit parasite expansion and mediate clearance. Consequently, blood stage infection is accompanied by a systemic pro-inflammatory immune response resulting in classical symptoms of mild malaria such as fever (Stevenson and Riley, 2004). However, some individuals progress to a severe course of malaria, partly owing to an imbalance in the pro- and anti-inflammatory immune response (Langhorne et al., 2008), resulting in malaria-associated mortality which is largely attributed to *P. falciparum* infections (WHO, 2016). One of the major complications of severe malaria is cerebral malaria (CM) which manifests with retinal abnormalities (Storm and Craig, 2014) as well as impaired consciousness or coma (Cunnington et al., 2013). The symptoms of CM are attributable to sequestration of infected erythrocytes and inflammatory leukocyte subsets, endothelial dysfunction, and inflammation (Storm and Craig, 2014), and these processes are mutually dependent and have synergetic effects (Cunnington et al., 2013). However, the precise molecular mechanisms underlying CM are not yet fully understood.

The induction of innate pro-inflammatory cytokine responses is mediated by germline-encoded pattern-recognition receptors, such as toll-like receptors (TLR), which recognize conserved microbial structures, i.e., pathogen-associated molecular patterns (PAMP) (Kawai and Akira, 2011). Among the malaria PAMP, glycosylphosphatidylinositols (GPI) are considered the main pathogenicity factor (Gowda, 2007). While GPI structure is conserved among *Plasmodium* species, human and *Plasmodium* GPI differ considerably (Boutlis et al., 2005). GPI serve as membrane anchors for certain cell surface proteins such as circumsporozoite protein and merozoite surface protein 1, and are also abundantly present free of protein attachment in membranes of pathogenic protozoa (Gowda, 2007; Gazzinelli et al., 2014). *P. falciparum* GPI have been found to induce the production of nitric oxide, tumor necrosis factor (TNF), and interleukin 1β (IL-1β) in murine macrophages *in vitro* (Schofield and Hackett, 1993; Tachado et al., 1996) and a synthetic malarial GPI glycan was demonstrated to be immunogenic *in vivo* (Schofield et al., 2002). Together, these findings point toward a role for *Plasmodium* GPI in malaria pathogenesis. Notably, *Plasmodium* GPI were described to be primarily recognized by TLR2 or heterodimers of TLR2/1 and TLR2/6 (Krishnegowda et al., 2005), yet TLR-deficiency did not protect mice from experimental cerebral malaria (ECM) (Togbe et al., 2007; Lepenies et al., 2008), indicating that TLR-mediated pro-inflammatory immune responses are not critical in the development of ECM. Since elucidating molecular mechanisms leading to malaria pathology might allow specific modulation of innate immune activation to prevent detrimental immune responses, this study was designed to identify potential *Plasmodium* GPI receptors. Using synthetic GPI affinity chromatography, we have identified the host protein moesin as an interaction partner of *P. falciparum* GPI and further addressed the functional relevance of this interaction in the development of malaria pathology. Moesin is a member of the ezrin-radixin-moesin (ERM) family of intracellular proteins which link actin filaments to transmembrane proteins (Louvet-Vallee, 2000) and interact with proteins involved in key signaling events, such as phosphatidylinositide 3-kinase, protein kinase A, or Rho-specific GDP dissociation inhibitors (Ivetic and Ridley, 2004; Niggli and Rossy, 2008; Ponuwei, 2016). Additionally, moesin cell surface translocation has been described upon lipopolysaccharide (LPS) stimulation *in vitro* (Iontcheva et al., 2004; Takamatsu et al., 2009), pointing toward a role for moesin in PAMP recognition. Moreover, we reasoned that moesin may play a role in the immune response to *Plasmodium* infection via its ability to interact with *Plasmodium* GPI as well as in malaria pathology due to its ability to modulate immunological synapse and endothelial paracellular gap formation (Itoh et al., 2002; Koss et al., 2006; Parameswaran and Gupta, 2013). Therefore, the capability of moesin to translocate to the cell surface upon *Plasmodium* GPI stimulation as well as the impact of moesin-deficiency on malaria PAMP-mediated cytokine induction and phagocytosis of *P. berghei* was analyzed *in vitro*. Additionally, *P. berghei* ANKA-infected moesin-deficient mice were used as a model to study the role of moesin in the host immune response to *Plasmodium* and in the development of cerebral pathology *in vivo*. We report here that despite the interaction between *Pf* GPI and moesin *in vitro*, moesin does not translocate to the cell surface in response to malaria PAMP in human and murine macrophages. Moreover, moesin-deficiency did not impair *Plasmodium*-induced cytokine responses *in vitro* and *in vivo* and did not protect mice infected with *P. berghei* ANKA from development of ECM.

## Materials and methods

### GPI affinity chromatography and mass spectrometry

GPI glycans were synthesized with a terminal sulfhydryl-containing linker (Kwon et al., 2005; Liu et al., 2005) to be covalently immobilized on SulfoLink® coupling gel (Pierce, Rockford, IL) according to the manufacturer's instructions. Using a syringe, the column was equilibrated by washing with coupling buffer (50 mM Tris, 5 mM EDTA, pH 8.5). Mouse macrophage cell-line RAW264.7 plasma membrane fraction was prepared as previously reported (Smart et al., 1995). Plasma membrane was solubilized in coupling buffer containing 0.5% Triton X-100. Samples were then loaded in the coupling buffer and the column was incubated for 1 h at 4°C. Non-bound and excess proteins were removed by washing the column with 3 column volumes of coupling buffer. Elution of bound proteins was carried out using 3 column volumes of coupling buffer containing 1 M mannose and 0.1% Triton X-100. Eluted protein extracts were subjected to 12.5% SDS-PAGE under non-reducing condition. After Coomassie staining, protein bands were excised, destained, and reduced prior to tryptic digestion and peptide mass fingerprinting. MALDI mass spectra were generated using a Voyager DE-STR MALDI-TOF MS system (PerSeptive Biosystems) with delayed extraction in the reflectron mode. Proteins were identified by comparison of peak lists generated from the Data Explorer application (PerSeptive Biosystems) against NCBInr (no redundant) and Swiss-prot databases using the Protein-Prospector V3.4.1 software MS-Fit (http://www.prospector.ucsf.edu).

### Moesin-GST expression and purification

A pGEX-4T-3 vector (Addgene) carrying Moesin-GST as N-terminal fusion protein was transformed into a bl21de3plysS (Promega, USA) *E. coli* strain. A single colony was picked to inoculate an overnight culture in 100 ml of LB medium supplemented with 100 μg/ml ampicillin at 37°C under shaking at 200 rpm. On the following day the overnight culture was diluted 1:20 in LB supplemented with 100 μg/ml ampicillin and incubated at 37°C at 300 rpm until an OD_600_ of 0.5 was reached. IPTG (Sigma-Aldrich) was added to the final concentration of 1 mM and the culture was incubated for additional 4 h. The bacteria pellet was harvested by centrifugation at 4,000 × g at 4°C for 20 min.

For the purification, bacteria pellet was resuspended in lysis buffer (50 mM Tris, 50 mM NaCl, 5 mM EDTA, 1 μg/ml leupeptin, 1 μg/ml pepstatin, 0.15 mM PMSF, 1 mM DFP, 1 mM 2-ME, pH 8.0) and lysis was completed by sonication. Cell lysate was centrifuged at 48,000 × g for 20 min at 4°C and the clear supernatant loaded on a GSH-agarose affinity column (Thermo Fisher). The column was washed three times to remove unbound material and moesin-GST was eluted with elution buffer (10 mM GSH in 50 mM Tris, 10 mM reduced glutathione, pH 8.0). The eluate was dialyzed against PBS and aliquots were kept at −80°C until use.

For surface plasmon resonance measurements, moesin was released from the moesin-GST fusion protein by thrombin digestion using immobilized thrombin, agarose (Sigma-Aldrich) according to the manufacturer's instruction (Supplementary Figure 1). Briefly, purified moesin was incubated with washed thrombin agarose beads in batch for 2 h at 37°C and the cleaved protein was recovered from the supernatant after centrifugation. Cleaved GST was removed by incubating the product from the thrombin cleavage with GSH-agarose beads in batch for 2 h at RT. Moesin without the GST tag was recovered in the supernatant after centrifugation of the beads at 10,000 × g for 10 min. Purified moesin was dialyzed against PBS and aliquots were frozen until use.

### Microarray binding assays

GPI microarrays were constructed as previously described (Ratner et al., 2004; Kamena et al., 2008) and covered with a FlexWell-64 (GRACE BIO-LABS, Bend, OR) layer to form a multi-well plate. Wells were then blocked with 5% milk powder in PBS for 1 h at RT followed by three washes with PBS containing 0.05% Tween-20. The wells were incubated with purified moesin-GST in PBS containing 0.05% Tween-20 and 0.5% BSA for 2 h at RT or with FITC-labeled concanavalin A (FITC-ConA) in ConA binding buffer (20 mM Tris; 500 mM NaCl; 1 mM CaCl_2_; 1 mM MgCl_2_; pH 7.4). After washing, the slides were incubated with rabbit polyclonal anti-GST antibodies for 1 h at RT. After extensive washing the slides were incubated 1 h at RT with ALEXA-Fluor® 594-labeled anti-rabbit secondary antibody (Invitrogen, Eugene. OR) at 1:1,000 in PBS containing 0.5% BSA and 0.05% Tween-20. The slides were then washed and fluorescence was revealed using an Affymetrix 427 laser scanner (MWG Biotech, Huntsville, AL).

### Surface plasmon resonance

**Figure 1:**
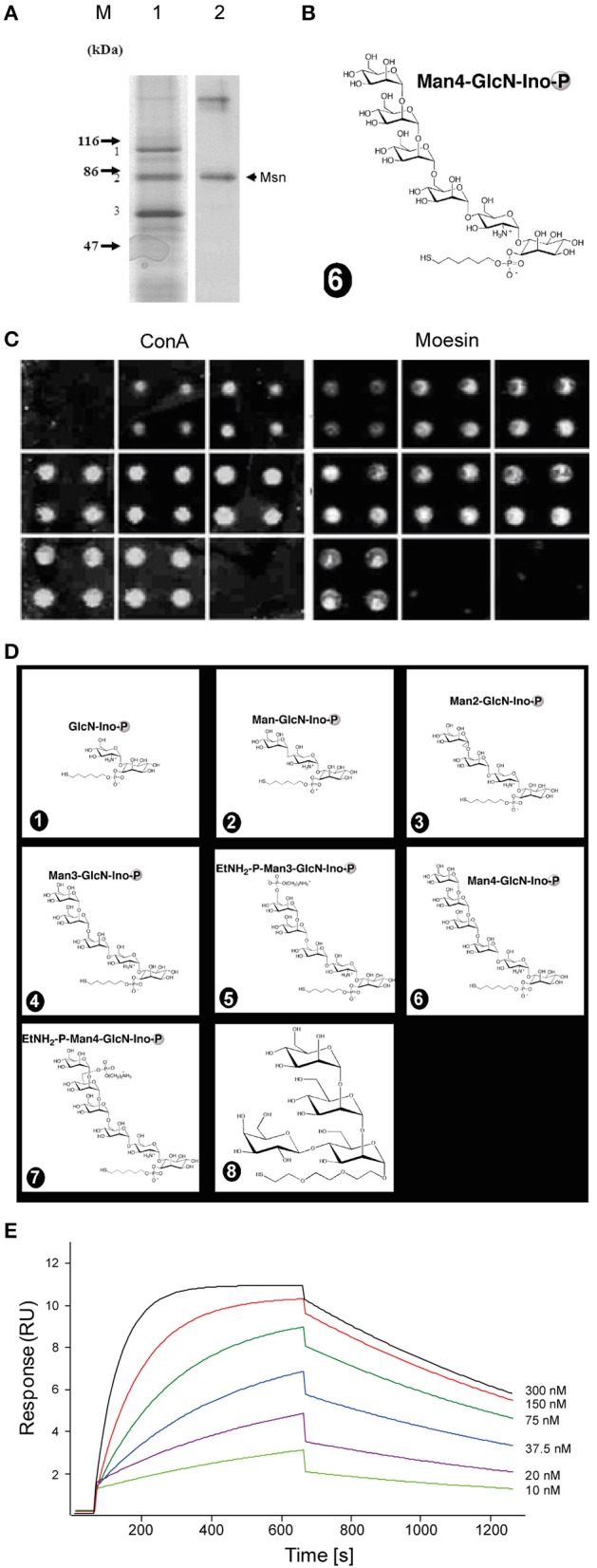
**Synthetic ***Plasmodium*** GPI glycan interacts with moesin. (A)** GPI-interacting proteins from an affinity chromatography were revealed by Coomassie staining on an SDS-PAGE **(1)** and after identification by mass spectrometry moesin was confirmed by western blot using anti-moesin antibody **(2)**. **(B)** The affinity column was generated using the full length glycan moiety of the GPI anchor. **(C,D)** Moesin interacts with GPI on microarray. Seven GPI glycan fragments and a polymannose control structure (depicted in D) were immobilized covalently as quadruplicate on glass slides to generate a microarray. **(C)** The slides were incubated with either FITC-ConA or GST-moesin. After incubation bound proteins were revealed by a fluorescent scanner. **(E)** Surface plasmon resonance measurement of moesin interaction with GPI glycan. Various concentrations of recombinantly expressed moesin (10, 20, 37.5, 75, 150, and 300 nM) were flown through the GPI-coupled gold chip and sensograms recorded and analyzed using the BIAevaluation software (BIAcore Life sciences) and the generated data were exported and graphs plotted using Origin 8.0 (OriginLab, Northampton) to obtain the K_*D*_ value. Double referenced association and dissociation phase data were globally fitted to a simple 1:1 interaction model (*A* + *B* = *AB*). The GPI glycan immobilized on the sensor chip was the same as that used for the affinity chromatography **(B)**.

GPI structure VI (Figure 1B) having a free thiol group was covalently attached to a gold surface CM5 chip using a Biacore T100 (GE Healthcare, Uppsala, Sweden). GPI glycan was immobilized on gold chips according to the manufacturer's protocol. Briefly, the carboxymethylated dextran matrix (CM5 chip) was activated at a flow rate of 10 μL/min using an 8 min injection pulse of an aqueous solution containing *N*-hydroxysuccinimide (NHS, 0.05 M) and *N*-ethyl-*N'*-(dimethylaminopropyl) carbodiimide (EDC, 0.2 M). The surface was further activated with a solution of *2*-(*2*-pyridinyldithio) ethanolamine (PDEA, 80 mM in 0.1 M sodium borate; pH 8.5) at the flow rate of 10 μL/min using a 10 min pulse. Next, a solution of synthetic GPI (50 μg/ml) containing 1 mM hexadecyltrimethylammonium chloride was flowed over the activated surface for 10 min at 4 μL/min. Remaining reactive groups on the surface were quenched by injection of a cysteine/NaCl solution (50 mM cysteine and 1 M NaCl in 0.1 M sodium acetate; pH 4.3) for 7.5 min at 10 μL/min. The reference flow cell was activated in parallel and ethanolamine was covalently attached. For K_*D*_ determination between immobilized GPI and moesin, HBS-EP buffer (10 mM HEPES, pH 7.4, 150 mM NaCl, 3 mM EDTA, 0.005% v/v surfactant P20) was used as running buffer. Various concentrations of moesin (10, 2, 37.5, 75, 150, and 300 nM) were injected into the flow cell for 10 min each at 20 μL/min at 25°C. After each sample, running buffer flowed over the sensor surface for 10 min to allow dissociation. The chip surface was regenerated for the next sample by injection of the regenerating solution (0.1% SDS, 0.085% H_3_PO_4_, 1 M NaCl and 0.1% HCl) for 1 min at 80 μL/min flow rate. The responses were calculated as the difference in response unit (RU) between analyte and reference flow cell and monitored as function of time (sensogram). Data processing and kinetic analysis was performed using the BIAevaluation software for T100 (Version 1.1.1) and graphs were plotted using Origin 8.0 (OriginLab, Northampton). Double referenced association and dissociation phase data were globally fitted to a simple 1:1 interaction model (*A* + *B* = *AB*).

To calculate the K_D_, the signal from the reference flow cell containing ethanolamine was subtracted from each value to correct for the contribution of non-specific interactions and systematic errors.

### THP-1 cells

The human monocytic leukemia cell line THP-1 (ATCC: TIB-202) was a kind gift from Dr. Pedro Moura-Alves (Max Planck Institute for Infection Biology, Berlin, Germany) and was cultured in RPMI 1640 (Gibco, Germany) supplemented with 10% fetal calf serum (FCS; Gibco), 2 mM L-glutamine (Gibco), 100 mM HEPES (Gibco), 1 mM sodium pyruvate (Sigma-Aldrich, Germany), 1% non-essential amino-acids (NEAA) (Gibco) and 55 μM β-mercaptoethanol (Sigma-Aldrich). For differentiation of THP-1 cells into macrophage-like cells, monocytic THP-1 cells were plated at a density of 0.6 × 10 e6 cells/ml in culture medium supplemented with 50–200 nM phorbol 12-myristate 13-acetate (PMA, Sigma-Aldrich) for 72 h (Moura-Alves et al., 2014). Upon transition of THP-1 cells into adherent growth, cells were washed with PBS repeatedly and rested in culture medium for 24 to 72 h. Successful differentiation was assessed in terms of expression of the cell surface marker CD11b by flow cytometry, since CD11b is not expressed on monocytic THP-1 cells (Schwende et al., 1996).

### Bone marrow-derived cells

Bone marrow-derived cells were isolated from C57BL/6 (Charles River Laboratories) or WT and moesin-deficient mice from the Max Planck Institute for Infection Biology breeding facility, according to Gonçalves and Mosser (2015). For the generation of bone marrow-derived macrophages (BMDM), bone marrow-derived cells were cultured in IMDM incomplete (IMDM (Gibco) containing 10% FCS and 2 mM L-glutamine) supplemented with 30% L-929 supernatant and 5% horse serum. Macrophage colony-stimulating factor (M-CSF)-expressing L-929 cells were kindly provided by Soo-Kyung Peuschel (Max Planck Institute for Infection Biology, Berlin, Germany). Medium was added at day 3 after seeding and a third of the volume was replaced at days 6 and 8 of culture. At day 10, differentiation was assessed by flow cytometry in terms of expression of macrophage surface markers CD11b and F4/80 as well as intracellular CD68 (Gonçalves and Mosser, 2015). Cells were re-plated for experiments or preserved in frozen stocks for future use.

For the generation of bone marrow-derived dendritic cells (BMDC), bone marrow-derived cells were further processed according to protocols adapted from Lutz et al. (1999), Brasel et al. (2000), Wells et al. (2005) and Madaan et al. (2014). Briefly, bone marrow-derived cells were resuspended in ACK lysis buffer (pH 7.2) and incubated for 2 min at 37°C for erythrocyte lysis. After washing, cells were cultured in RPMI 1640 supplemented with 10% FCS, 1% NEAA, 2 mM L-glutamine, 100 U/ml penicillin/streptomycin (Gibco), 50 μM β-mercaptoethanol, and 20 ng/ml granulocyte-macrophage colony-stimulating factor (GM-CSF; Miltenyi Biotec, Germany) as well as 20 ng/ml IL-4 (Miltenyi Biotec). After 3 days of culture, fresh medium was added to the cells, and half of the medium was replaced with fresh medium at days 6 and 8. At day 10, loosely adherent cells were collected and re-plated in culture medium without IL-4 and 10 ng/ml GM-CSF for 24 h. Optionally, BMDC were LPS-primed (100 ng/ml) for 24 h to mature. BMDC differentiation was assessed by flow cytometry in terms of expression of DC surface markers CD11b, CD11c, and MHCII, as well as F4/80. Upon successful differentiation, BMDC were re-plated for stimulation experiments.

### Cell stimulation

Cells were stimulated with 10 ng/ml LPS (LPS from *E. coli* 0111:B4, Sigma-Aldrich), 10 ng/ml TNF (Miltenyi Biotec, Germany), *P. falciparum* schizont extract diluted 1:100 in culture medium, *P. berghei* schizont extract diluted 1:1,000 in culture medium, or *P. berghei* schizonts added at a ratio of 1:10, i.e., 10 schizonts per cell. *P. falciparum* schizonts were obtained through a percoll gradient centrifugation of a mixed-stages culture as previously described (Rivadeneira et al., 1983). Purified schizonts were washed in PBS and subsequently sonicated to produce schizont extract. *P. berghei* schizont extract was generated by repeated freeze-thaw cycles and adjusted to contain the extract of 4 × 10 e6 schizonts/μl in PBS.

### Schizont culture

For schizont enrichment, blood of *P. berghei*-infected mice was cultured in RPMI 1640 supplemented with 20% FCS (Gibco) and 15 μg/ml gentamycin (Gibco) with mild shaking for 20–24 h at 37°C and 80% humidity under 5% O_2_ and 5% CO_2_ (Matz et al., 2015). Schizont development was assessed by Giemsa-stained thin blood smear and schizonts were purified by density gradient centrifugation using 60% Percoll (GE Healthcare, UK) in PBS. Schizonts were collected from the interphase, washed repeatedly and further processed depending on experimental end-point.

### Transcript quantification

RNA was isolated with TRIzol reagent (Ambion, Germany), glycogen (Ambion) and ammoniumacetate (Ambion) according to the manufacturer's instructions. Complementary DNA (cDNA) was synthesized from 1 μg total RNA per sample by reverse transcription using the RETROscript reverse transcription kit (Ambion) according to the manufacturer's instructions. Transcript abundance was determined by qPCR with reactions carried out on a StepOne Plus (Applied Biosystems, Germany) with Power SYBR green master mix (Applied Biosystems) and Quantitect primer assays (Qiagen, Germany) for the following transcripts: *Ppib* (Mm_Ppib_1_SG), *Tnf* (Mm_Tnf_1_SG), *Il1b* (Mm_Il1b_2_SG), *Il12a* (Mm_Il12a_1_SG), *Ifng* (Mm_Ifng_1_SG), as well as with primers Hs_*RPL13A*_fwd 5′-CCTGGAGGAGAAGAGGAAAGAGA-3′, Hs_*RPL13A*_rev 5′-TTGAGGACCTCTGTGTATTTGTCAA-3′ (Rizopoulos et al., 2016), Mm_*Gapdh*_fwd 5′-TGAGGCCGGTGCTGAGTATGTCG-3′, Mm_*Gapdh*_rev 5′-CCACAGTCTTCTGGGTGGCAGTG-3′ (Sato et al., 2014) (synthesized by Eurofins Genomics, Germany). qPCR was performed in technical triplicates with the following cycling conditions: 94°C for 15 min and 40 cycles of 94°C for 15 s, 60°C for 60 s. For determining phagosomal degradation, *P. berghei* 18 s RNA transcripts were amplified as specified in Friesen et al. (2010). Melt curve analysis was included in each run to verify the specificity of each reaction. Relative transcript abundance and fold change were determined using the comparative threshold cycle (C_T_) method (Schmittgen and Livak, 2008), while *RPL13A, Gapdh*, or *Ppib* served as internal controls.

### Western blot

For the detection of proteins using Western blot, samples were prepared by resuspension in sample buffer (Laemmli 2x concentrate, Sigma-Aldrich), separated by SDS-PAGE, and transferred onto Amersham Hybond P 0.45 PVDF membranes (GE Healthcare). Membranes were incubated with anti-MSN antibody (clone 38/87, Sigma-Aldrich; or clone EP1863Y, Abcam, UK) or anti-GAPDH antibody (clone 71.1, Sigma-Aldrich) as a loading control. Antibodies were detected using corresponding anti-mouse or anti-rabbit antibody coupled to HRP (Jackson Immunoresearch, UK) and ECL Western blotting substrate (Pierce, Germany). PageRuler pre-stained protein ladder (Fermentas) was used to determine the molecular weight of separated proteins.

### Quantification of cytokines and chemokines

THP-1, BMDM, and BMDC supernatants as well as serum of *P. berghei* ANKA Bergreen-infected mice were assayed for cytokines by human or mouse TNF DuoSet ELISA (R&D systems, USA) in combination with the DuoSet ELISA ancillary reagent kit (R&D systems) or by cytometric bead array for the murine cytokines TNF, IFN-γ, IL-6, IL-10, IL-12p70, and MCP-1/CCL2 by using the CBA mouse inflammation kit (BD Biosciences, Germany). Each kit was used according to the manufacturer's instructions.

### Flow cytometry

Cells were incubated with human or mouse FcR blocking reagent (Miltenyi Biotec) according to the manufacturer's instructions prior to antibody staining. Cells were stained with the following antibodies: anti-human CD11b (ICRF44, eBioscience, Germany), anti-mouse CD11b (M1/70, eBioscience), CD11c (N418, eBioscience), CD68 (FA-11, eBioscience), F4/80 (BM8, eBioscicence), MHCII (M5/114.15.2, eBioscience), anti-Moesin (38/87, Sigma-Aldrich; EP1863Y, Abcam), anti-mouse IgG1 (M1-14D12, eBioscience) or anti-rabbit IgG (poly4064, Biolegend; polyclonal, Invitrogen). Stainings included fixable viability dyes LIVE/DEAD fixable dead cell stain aqua (Invitrogen) or fixable viability dye eFluor780 (eBioscience). Fixable viability dyes and antibodies for cell surface antigens were diluted in PBS and added to cells for 15 min at 4°C. For staining of intracellular proteins, cells were fixed in 2% paraformaldehyde (PFA, Sigma-Aldrich) and permeabilized using Permeabilizing solution 2 (BD Biosciences). Antibodies for intracellular antigens were diluted in PBS and added to cells for 30 min at 4°C. For unconjugated primary antibodies, a further incubation step with a secondary fluorochrome-conjugated antibody was performed (30 min, 4°C). Cells were acquired on a FACSCanto or Fortessa (BD Biosciences) in the flow cytometry core facility of the Deutsches Rheuma-Forschungszentrum and data was analyzed in FlowJo (Treestar, USA). The gating strategy employed excluded doublets and dead cells from the analysis and further gates were set based on fluorescence-minus-one (FMO) and unstained controls.

### Phagocytosis assay

BMDM were incubated with labeled or unlabeled P. *berghei* ANKA Bergreen parasites expressing GFP under the HSP70 promoter (Kooij et al., 2012). Briefly, *P. berghei* ANKA Bergreen schizonts were isolated by density gradient centrifugation and stored in 10% glycerol (Roth) in Alsever's solution (Sigma-Aldrich) at −80°C. Due to freeze-thawing of schizonts, merozoites were released from erythrocytes, subsequently washed with PBS and labeled according to a protocol adapted from Cambos and Scorza (2011). Briefly, merozoites were labeled with 5 μM caroboxyfluorescein diacetate succinimidyl ester (CFDA-SE; Molecular Probes, Germany) or 2.5 μM CellTrace Violet (Molecular Probes) in PBS supplemented with 0.1% FCS for 3 min at room temperature. Residual dye was diluted with 10%FCS in PBS, followed by an additional washing step with 10%FCS in PBS. CFSE- or CellTrace Violet-labeled merozoites were resuspended in BMDM culture medium and added to cells at a ratio of 1:10, i.e., 10 merozoites per one BMDM. Alternatively, *P. berghei* ANKA Bergreen schizonts were isolated and immediately added at a ratio of 1:10 to BMDM without prior labeling. At indicated time points, cells were collected on ice, washed in PBS, and stained for cell surface markers or immediately resuspended in acquisition buffer. Phagocytosis was analyzed by flow cytometry based on detection of CFSE, CellTrace Violet, or GFP for unlabeled schizonts. Gate settings were determined using untreated BMDM.

### Ethics statement

All animal work was conducted in accordance with the German “Tierschutzgesetz in der Fassung vom 22. Juli 2009,” which implements Directive 2010/63/EU from the European Parliament and Council (On the Protection of Animals Used for Scientific Purposes). The protocol was approved by the ethics committee of the Berlin state authorities (LaGeSo).

### Mice

C57BL/6 and NMRI mice were obtained from Charles River laboratories (Germany). Moesin-deficient (Doi et al., 1999) and corresponding wild type control colonies on a C57BL/6 background were maintained by the Max Planck Institute for Infection Biology breeding facility. SNP genotyping (Taconic, USA) revealed that moesin-deficient mice were backcrossed to the C57BL/6 background for at least six generations. Moesin-deficient mice were identified by PCR using *Taq* DNA polymerase (Fermentas) and standard cycling conditions with primers *Msn*_fwd1 5′-CTGAAGTCGGACAAAGATTTCCAGG-3′, *Msn*_fwd2 5′-CATCAGTATATGAAACAGCCCCCTG-3′, *Msn*_rev 5′-AGGTGTCTCCCAGAGATACGATTTGG-3′ (synthesized by Eurofins Genomics). Mice were kept under specific pathogen-free conditions with *ad libitum* diet at the Max Planck Institute for Infection Biology animal facility in a 12 h light/12 h dark cycle.

### *P. berghei* ANKA bergreen *In vivo* infection

Six to nine week old female *Msn-/-* or WT mice were infected with 10,000 *P. berghei* ANKA Bergreen-infected erythrocytes derived from an NMRI donor mouse by i.v. injection. Parasitemia was determined daily by flow cytometry starting day 3 post-infection. Briefly, blood of infected mice was diluted in Alsever's solution containing Hoechst 33342 (Invitrogen) and acquired on a Fortessa flow cytometer (BD Biosciences). Parasitemia was assessed by determining the fraction of GFP^+^ Hoechst 33342^+^ cells in the samples. Blood from a naïve mouse served as control for determination of gate settings. Cells single positive for Hoechst 33342, representing leukocytes, were excluded from the analysis. Additionally, serum was collected by retrobulbar punction at indicated time points. Mice were closely monitored for behavioral symptoms of ECM, such as convulsions, absence of touch escape, and unconsciousness (Lackner et al., 2006), and sacrificed when presenting with severe neurological impairment.

### Data analysis

All data was imported into Prism 7.0 (GraphPad) for statistical analysis. Statistical analyses to determine the *P-*value of each comparison are indicated individually. No statistical analysis was performed on data sets with *n* < 3.

## Results

### Moesin interacts with *Pf*GPI

The GPI of *Plasmodium* origin has been widely described to induce pro-inflammatory responses from host immune system via pattern recognition receptors such as TLR2 and TLR4 (Krishnegowda et al., 2005). However, observations that TLR2/4 double knockout mice show no resistance to cerebral malaria (Togbe et al., 2007) prompted the question whether additional receptors might be involved in this process. In order to look for interaction partners of *Pf* GPI, we immobilized chemically synthesized GPI glycan (Figure 1B) on an affinity column and performed pull-down experiments using plasma membrane preparation from the mouse macrophage cell line RAW264.7. Proteins bound to synthetic GPI glycan were eluted from the column, subjected to SDS-PAGE and identified after trypsin digestion by peptide mass fingerprinting. The membrane-organizing extension spike protein moesin was identified as one of the most prominent GPI-binding protein, and was confirmed by western blot using anti-moesin antibody (Figure 1A). Other proteins were also identified but were mostly very abundant proteins that might easily result from contamination such a keratin, actin, and histones. Additionally, proteins such as CD1b were identified along with moesin as potential binding candidates. However, since moesin showed the highest probability after repeated experiments and given the postulated role in LPS signaling (Tohme et al., 1999; Amar et al., 2001; Iontcheva et al., 2004; Zawawi et al., 2010), moesin was selected for further investigations.

### Moesin binds to *Pf*GPI on microarray and on surface plasmon resonance (SPR)

In a first attempt to confirm the interaction between moesin and GPI glycans, a carbohydrate microarray containing seven synthetic GPI glycans of different length (Figure 1D positions 1 to 7), as well as a cap polysaccharide from *Leishmania* as control (Figure 1D position 8) was constructed. Incubation of recombinantly expressed GST-moesin with the carbohydrate array revealed binding of moesin to all GPI fragments but not to the cap polysaccharide. In a control experiment, the mannose-specific lectin concanavalin A bound all mannose-containing GPI fragments as well as the cap polysaccharide, but not the shortest GPI fragment lacking any mannose (Figure 1C). Next, we studied the kinetics of the interaction between moesin and *Pf* GPI using surface plasmon resonance (SPR). The full GPI-glycan of *Plasmodium falciparum* (Figure 1B) was covalently immobilized on an SPR gold chip. Binding was assessed using various concentrations of purified moesin. The result showed a linear concentration-dependent binding of moesin to GPI-glycan (Figure 1E). Strikingly, the binding constant (K_D_) for the interaction between moesin and GPI was 9.7 × 10^−4^ M, which is relatively weak but is in the typical range for carbohydrate interaction with carbohydrate-binding proteins (Goldstein and Poretz, 1986; Lee and Lee, 1995). Taken together these results clearly indicated an interaction between moesin and the carbohydrate moiety of the *Pf* GPI anchor.

### Malaria PAMP do not induce moesin cell surface translocation on macrophage-like THP-1 cells

**Figure 2:**
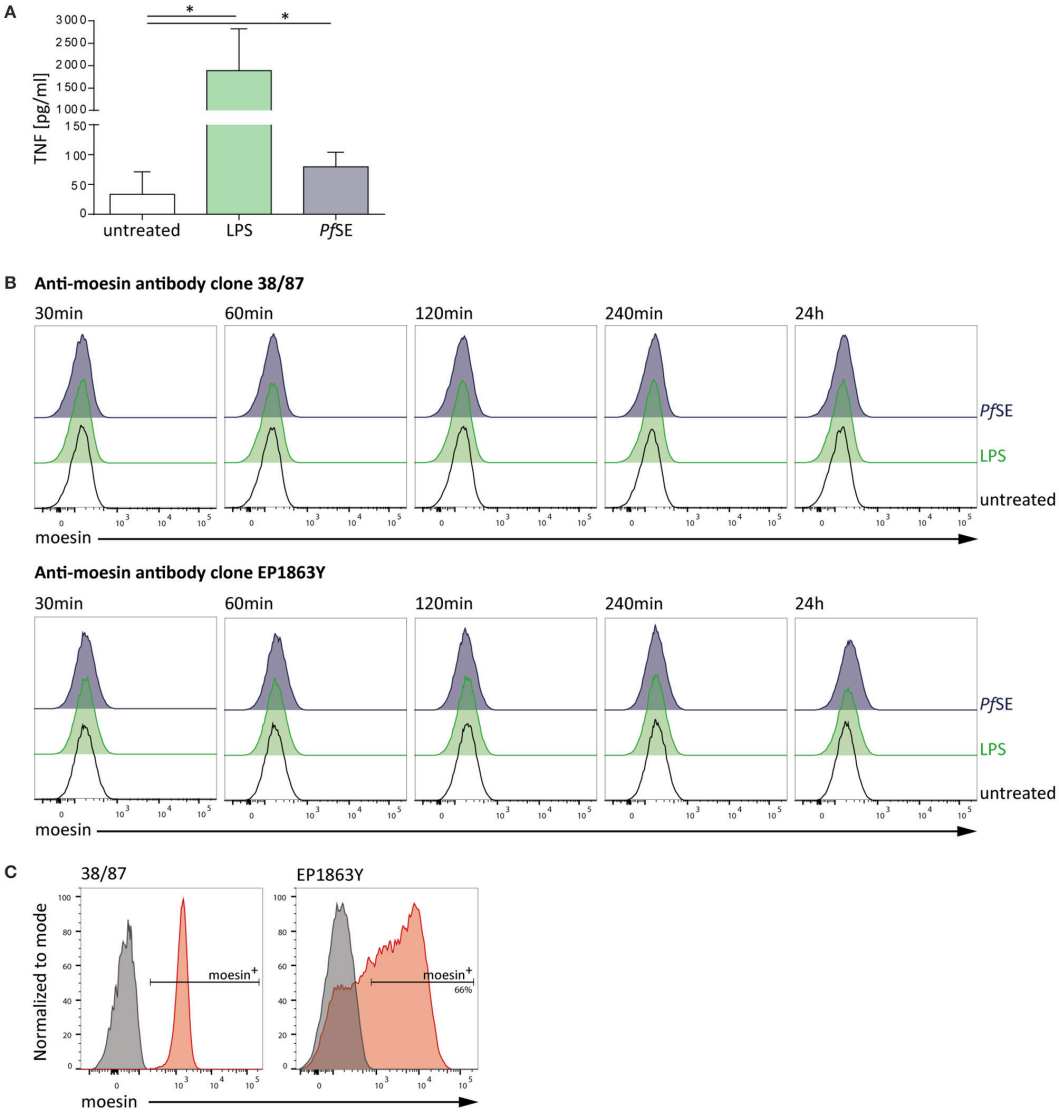
**Macrophage-like THP-1 cell response to stimulation is not accompanied by moesin cell surface localization. (A)** Macrophage-like THP-1 cell responsiveness to stimulation determined by TNF-ELISA after 24 h treatment; LPS: 10 ng/ml, *P. falciparum* schizont extract (*Pf*SE): 1:100 in medium; *n* = 5, bars indicate mean + SD; One-way ANOVA of paired samples with Dunnett correction revealed ^*^P < 0.05. **(B)** Representative histograms of moesin cell surface staining in macrophage-like THP-1 cells at different time points of stimulation, detected by flow cytometry with time points and treatments indicated, gated on viable CD11b^+^ cells, detection with anti-moesin antibody clone 38/87, and anti-mouse IgG-FITC [upper panel; cells differentiated with 20 ng/ml PMA for 24 h (Iontcheva et al., 2004))] and with anti-moesin antibody clone EP1863Y and anti-rabbit IgG-PE (lower panel; cells differentiated with 200 nM PMA for 72 h) **(C)** Intracellular staining control for moesin; left: viable undifferentiated THP-1, right: viable differentiated THP-1; gray: secondary antibody staining control, red: moesin^+^ cells detected with anti-mouse IgG-APC for clone 38/87, and anti-rabbit IgG-PE for clone EP1863Y. Dead cells were excluded from all flow cytometric analyses by using a fixable viability dye.

After clearly establishing the binding of moesin to *Pf* GPI we next sought to determine the physiological relevance of this interaction in the context of malaria. The classification of *Pf* GPI as one of the major malaria toxins is due to its ability to induce the secretion of pro-inflammatory cytokines from host innate immune cells such as macrophages *in vitro* (Schofield and Hackett, 1993; Tachado et al., 1996; Krishnegowda et al., 2005; Zhu et al., 2009). Intriguingly, moesin is primarily described as an intracellular protein (Ponuwei, 2016), yet translocation to the cell surface of e.g., macrophage-like THP-1 cells was reported to be induced by LPS stimulation (Iontcheva et al., 2004). Consequently, it was hypothesized that moesin might acts as a macrophage cell surface receptor for *Plasmodium* GPI. Given that GPI are abundantly present on the parasite surface (Gowda, 2007), preparations of schizont extracts were used as a stimulus containing *Plasmodium* GPI. In order to evaluate the responsiveness of human macrophage-like THP-1 cells to stimulation with LPS or GPI-containing *P. falciparum* schizont extract (*Pf* SE), TNF secretion was determined in cell culture supernatants by ELISA. While macrophage-like THP-1 cells secrete TNF to a great extent when stimulated with LPS, stimulation with *Pf* SE also results in a consistent yet less pronounced induction of TNF secretion from differentiated THP-1 cells (Figure 2A). Given the suitability of the stimuli applied to elicit a pro-inflammatory response, next we assessed the cell surface localization of moesin upon stimulation of human macrophage-like THP-1 cells with LPS or *Pf* SE by flow cytometry. Surprisingly, moesin cell surface translocation was not detected on viable differentiated THP-1 cells with anti-moesin antibody 38/87 in either LPS- or *Pf* SE-treated samples at all timepoints tested (Figure 2B). This finding was further corroborated by using another anti-moesin antibody clone (EP1863Y) (Figure 2B). Moreover, the suitability of the staining protocol was verified by successful intracellular moesin detection with each anti-moesin antibody clone (Figure 2C). Taken together, these results indicate that even though macrophage-like THP-1 cells respond to both LPS and *Pf* SE by TNF secretion, this response is not accompanied by recruitment of moesin to the cell surface.

### Macrophage response to malaria PAMP is moesin-independent

Since *Pf* GPI-induced TNF secretion was reported to be MyD88-dependent (Krishnegowda et al., 2005), another aspect to be investigated by this study was the role of moesin in *Plasmodium* GPI-induced signal transduction leading to the secretion of pro-inflammatory cytokines. In order to address this question *in vitro*, we isolated cells from moesin-deficient mice (Doi et al., 1999). Moesin-deficient mice were identified by PCR (Supplementary Figure 2A) and lack of moesin protein was validated via western blot. Surprisingly, use of anti-moesin antibody 38/87 suggested the presence of moesin in cells isolated from moesin-deficient mice (Supplementary Figure 2B), while anti-moesin antibody EP1863Y confirmed the absence of moesin from moesin-deficient mice (Supplementary Figure 2C). Since anti-moesin antibody 38/87 recognizes a second, slightly heavier protein in human THP-1 cells (Supplementary Figure 2B), and the protein recognized in murine samples corresponds to the size of the upper band detected in human THP-1 cells, it may be concluded that this antibody clone binds unspecifically to a protein other than moesin in murine samples. Consequently, anti-moesin antibody clone EP1863Y was used for subsequent experiments. Next, bone marrow-derived macrophages (BMDM) were generated from both wild type (WT) and moesin-deficient C57BL/6 mice. Successful differentiation was confirmed by flow cytometric analysis of the key mouse macrophage surface markers CD11b and F4/80, as well as intracellular CD68 (Gonçalves and Mosser, 2015). All viable WT and moesin-deficient BMDM expressed both, CD11b and F4/80, and cells were also largely positive for CD68, thus verifying efficient differentiation of bone marrow-derived cells (Supplementary Figure 2D).

**Figure 3:**
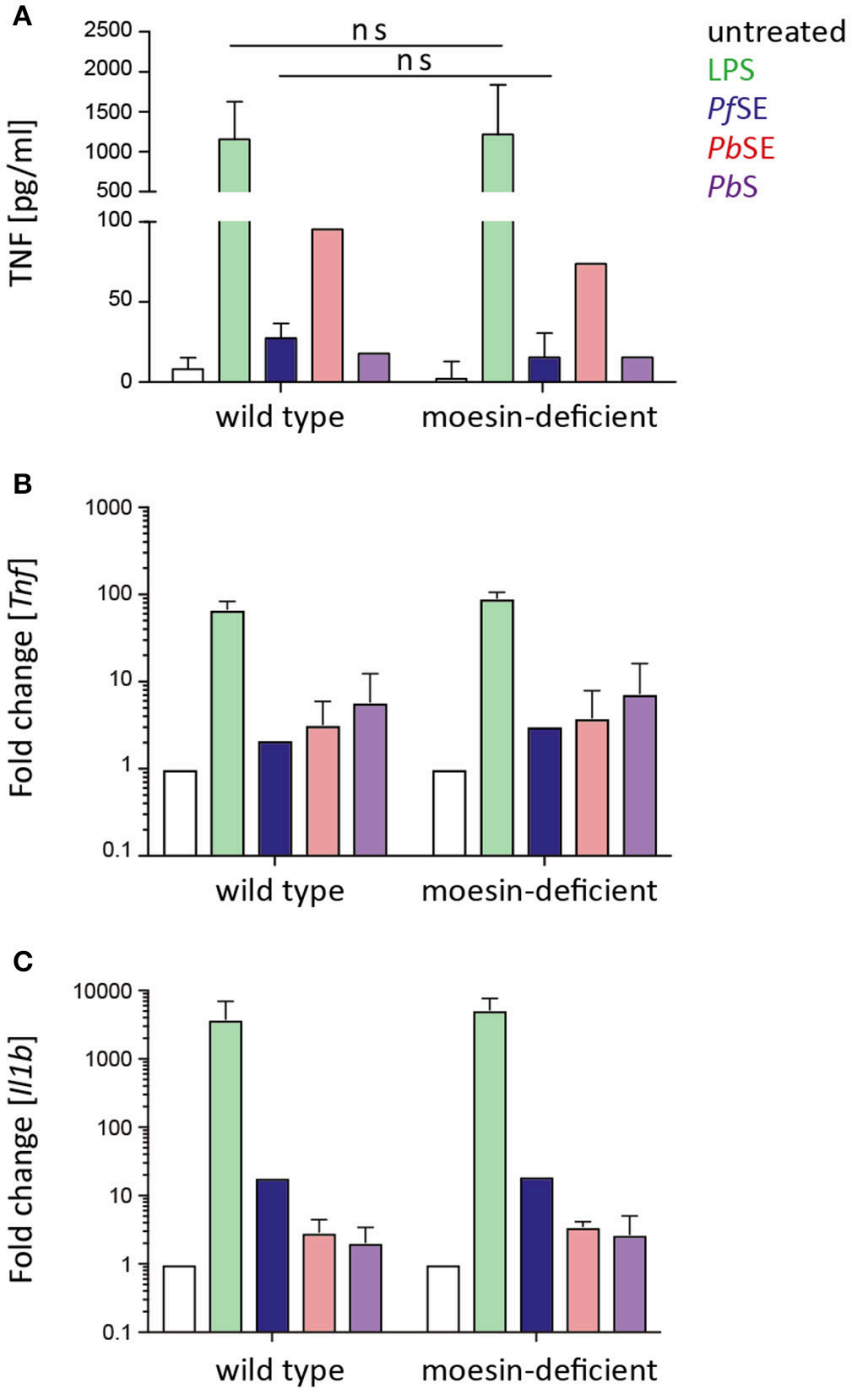
**BMDM response to stimulation is moesin-independent. (A)** TNF secretion upon BMDM stimulation determined in supernatants after 24 h stimulation by TNF-ELISA and CBA, *n* = 4 for untreated/LPS/*P. falciparum* schizont extract (*Pf*SE), *n* = 1 for *P. berghei* schizont extract (*Pb*SE), and *P. berghei* schizonts (*Pb*S); unpaired *t*-test revealed that differences are not significant (ns); **(B,C)** *Tnf* and *Il1b* expression detected by qPCR, relative expression was calculated with respect to *Gapdh*, relative expression in treated groups was normalized to untreated cells; *n* = 2 for LPS/*Pb*S/*Pb*SE, *n* = 1 for *Pf*SE; LPS: 10 ng/ml, *Pf*SE: 1:100 dilution in medium, *Pb*SE: 1:1,000 dilution in medium, *Pb*S added at a ratio of 1:10. Bars indicate mean + SD.

Upon confirmation of differentiation of WT and moesin-deficient BMDM, cells were stimulated with LPS, *Pf* SE, *P. berghei* schizont extract (*Pb*SE), and *P. berghei* schizonts (*Pb*S) for 24 h and cell culture supernatants were analyzed for TNF concentrations by ELISA or CBA. Despite previous reports on markedly reduced LPS-induced TNF secretion in the absence of moesin (Iontcheva et al., 2004) or antibody-mediated moesin blocking (Tohme et al., 1999; Amar et al., 2001; Zawawi et al., 2010), TNF concentration in supernatants of LPS-treated moesin-deficient BMDM was similar to WT BMDM in the experimental setting used here (Figure 3A). Additionally, the TNF response induced by *Plasmodium*-derived stimuli was not markedly different between WT and moesin-deficient BMDM. Since the TNF response induced by malaria PAMPs was generally low and variable for both WT and moesin-deficient cells, potential differences in TNF secretion may not be detectable in this experimental setting. Therefore, transcript levels of *Tnf* and *Il1b* were determined by qPCR in both WT and moesin-deficient BMDM after 4 h of stimulation under otherwise unchanged conditions. In good agreement with TNF concentration in BMDM supernatants, induction of TNF transcripts was most pronounced in LPS-treated samples (~100-fold compared to untreated BMDM). Transcription of the *Tnf* gene was also induced by *Plasmodium*-derived stimuli, yet to a much lower extent (~5-fold compared to untreated BMDM) than that initiated by LPS treatment (Figure 3B). Nevertheless, induction of TNF transcripts was similar in WT and moesin-deficient BMDM for all conditions tested. Another *Plasmodium* GPI-induced cytokine is IL-1β (Schofield and Hackett, 1993), and LPS-induced secretion of IL-1β has also been demonstrated to be moesin-dependent (Zawawi et al., 2010), thus indicating a role for moesin in signal transduction leading to IL-1β secretion. However, in the present study, induction of *Il1b* gene transcription did not markedly differ in WT and moesin-deficient BMDM for all stimuli applied, including LPS (Figure 3C). Taken together, these results demonstrate that the macrophage pro-inflammatory response to malaria PAMPs in terms of TNF secretion as well as *Tnf* and *Il1b* gene transcription is not as pronounced as that elicited by LPS. Furthermore, moesin-deficient and WT BMDM responded similarly to LPS and *Plasmodium*-derived stimuli, suggesting that moesin is not essential in signaling processes leading to TNF secretion and *Il1b* gene transcription in murine BMDM.

**Figure 4:**
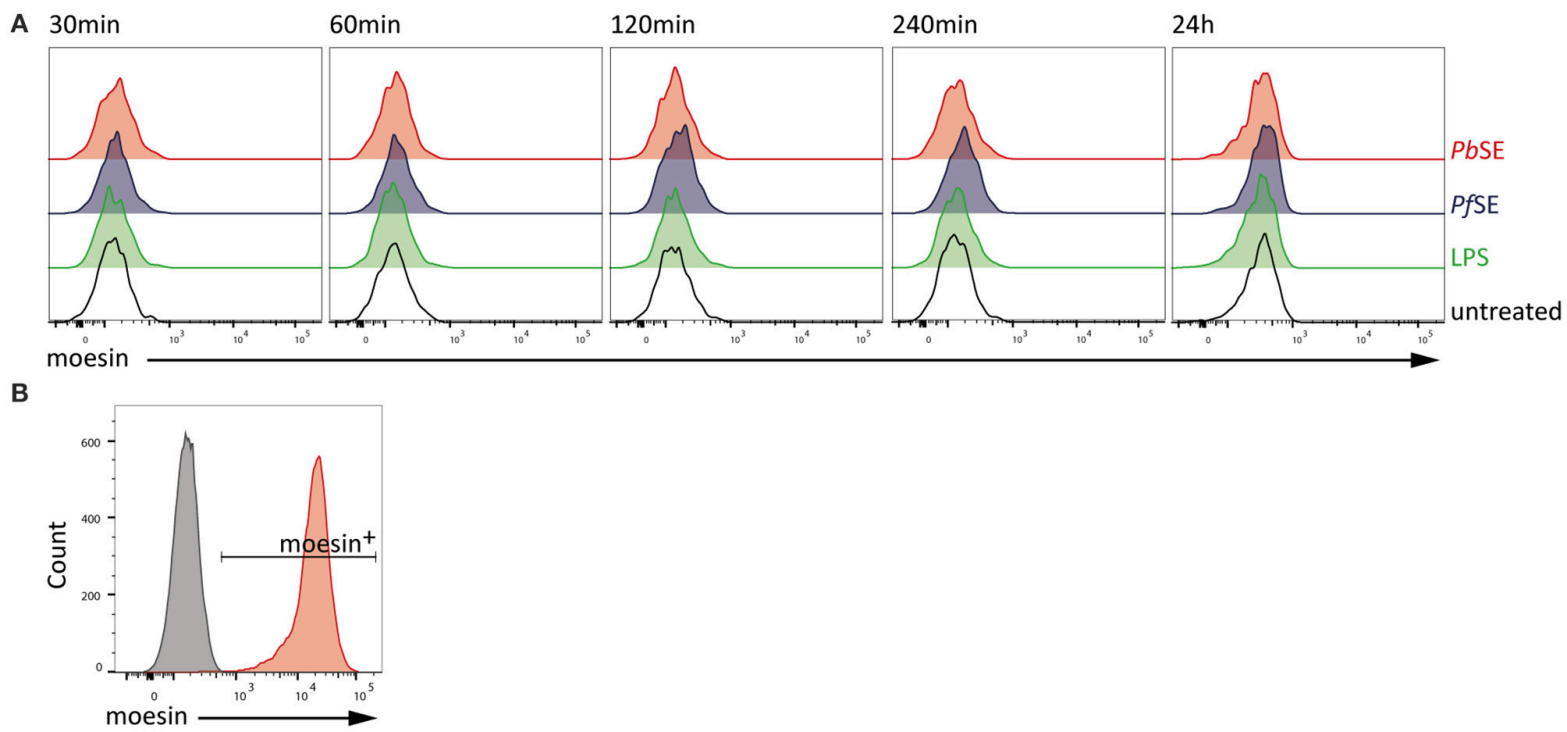
**Absence of moesin on BMDM cell surface. (A)** Flow cytometric analysis of moesin cell surface localization on viable CD11b^+^F4/80^+^ BMDM over time with stimuli indicated; detection of anti-moesin EP1863Y using anti-rabbit-PE; **(B)** Intracellular moesin detection in viable CD11b^+^ BMDM; gray: secondary antibody control, red: moesin^+^ cells using anti-moesin antibody EP1863Y and anti-rabbit IgG-AlexaFluor488.

Since it cannot be excluded that stimulation-induced cell surface localization of moesin is required for prompting other processes so far not covered in this study, we also investigated the translocation of moesin to the cell surface of macrophages upon stimulation with PAMPs in the murine system. Therefore, WT BMDM were stimulated with LPS, *Pf* SE, and *Pb*SE for different periods of time and analyzed for moesin cell surface expression via flow cytometry. In line with our observations in differentiated human THP-1 cells, localization of moesin to the cell surface of viable WT BMDM was not detected with anti-moesin antibody clone EP1863Y in either LPS-, *Pf* SE-, or *Pb*SE-treated samples at all timepoints tested (Figure 4A), while the intracellular staining control demonstrated the suitability of the protocol to detect moesin (Figure 4B).

### Moesin-deficient macrophages display unaltered phagocytic activity

An alternative site for moesin and *Plasmodium* GPI to interact is at the phagosomal membrane (Desjardins et al., 1994; Defacque et al., 2000) and thus moesin might orchestrate parasite recognition and/or degradation (Defacque et al., 2000; Erwig et al., 2006). Consequently, experiments aimed at investigating whether moesin is critically involved in phagocytic uptake and degradation of *P. berghei* merozoites or schizonts were performed.

**Figure 5:**
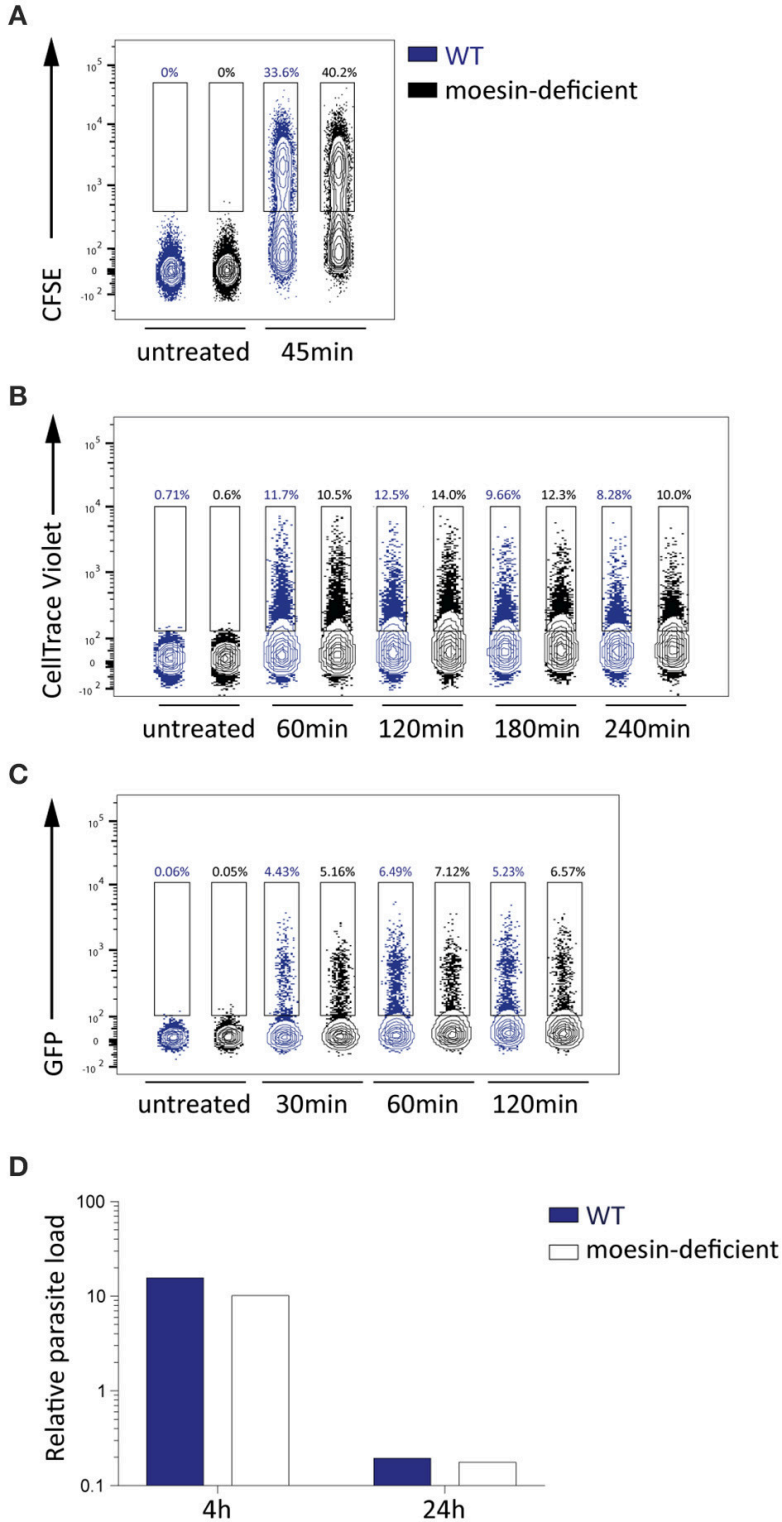
**Phagocytosis and phagosomal degration of parasites in BMDM is moesin-independent. (A–C)** Flow cytometric analysis of *P. berghei* phagocytosis by WT and moesin-deficient BMDM for the time indicated, numbers represent percentage of BMDM that phagocytosed parasites; *n* = 1 for all sets **(A)** Phagocytosis of CFSE-labeled *P. berghei* ANKA merozoites in viable CD11b^+^ F4/80^+^ BMDM; **(B)** Phagocytosis of CellTrace Violet-labeled *P. berghei* ANKA merozoites in BMDM; **(C)** Phagocytosis of unlabeled *P. berghei* ANKA Bergreen (GFP^+^) schizonts in BMDM; **(D)** Detection of *P. berghei* 18 s rRNA in BMDM by qPCR, expressed as relative parasite load, after 4 and 24 h of incubation with *P. berghei* ANKA merozoites (*n* = 1), reduction in *P. berghei* 18 s rRNA indicates phagosomal degradation.

In order to determine the impact of moesin-deficiency on *P. berghei* merozoite internalization, CFSE- or CellTrace Violet-labeled *P. berghei* merozoites were added to WT and moesin-deficient BMDM for different periods of time and phagocytosis was analyzed by flow cytometry (Figures 5A,B). Since the two approaches revealed comparable phagocytic uptake of *P. berghei* merozoites by WT and moesin-deficient BMDM, unlabeled *P. berghei* schizonts expressing high levels of GFP [ANKA Bergreen; (Kooij et al., 2012)] were used next in order to exclude that excess CFSE or CellTrace Violet dye resulted in BMDM labeling instead of reflecting phagocytosis. Although the three approaches differed in overall detection levels of phagocytic activity, phagocytic uptake of parasites was consistently similar in WT and moesin-deficient BMDM within experiments at all-time points tested (Figures 5A–C). Additionally, WT and moesin-deficient BMDM were incubated with *P. berghei* merozoites for 4 and 24 h in order to assess parasite degradation on the RNA level via qPCR. In good agreement with the data obtained by flow cytometry, *P. berghei* rRNA levels were similar in WT and moesin-deficient BMDM after 4 h of incubation (Figure 5D), thus confirming that moesin is not essential for phagocytic uptake of *P. berghei* merozoites. Additionally, *P. berghei* rRNA levels were markedly reduced after 24 h of incubation in both WT and moesin-deficient BMDM to a similar extent (Figure 5D), thereby indicating that lack of moesin does not impair phagosomal degradation of parasite material.

### Moesin deficiency does not affect dendritic cell response to malaria PAMP

**Figure 6:**
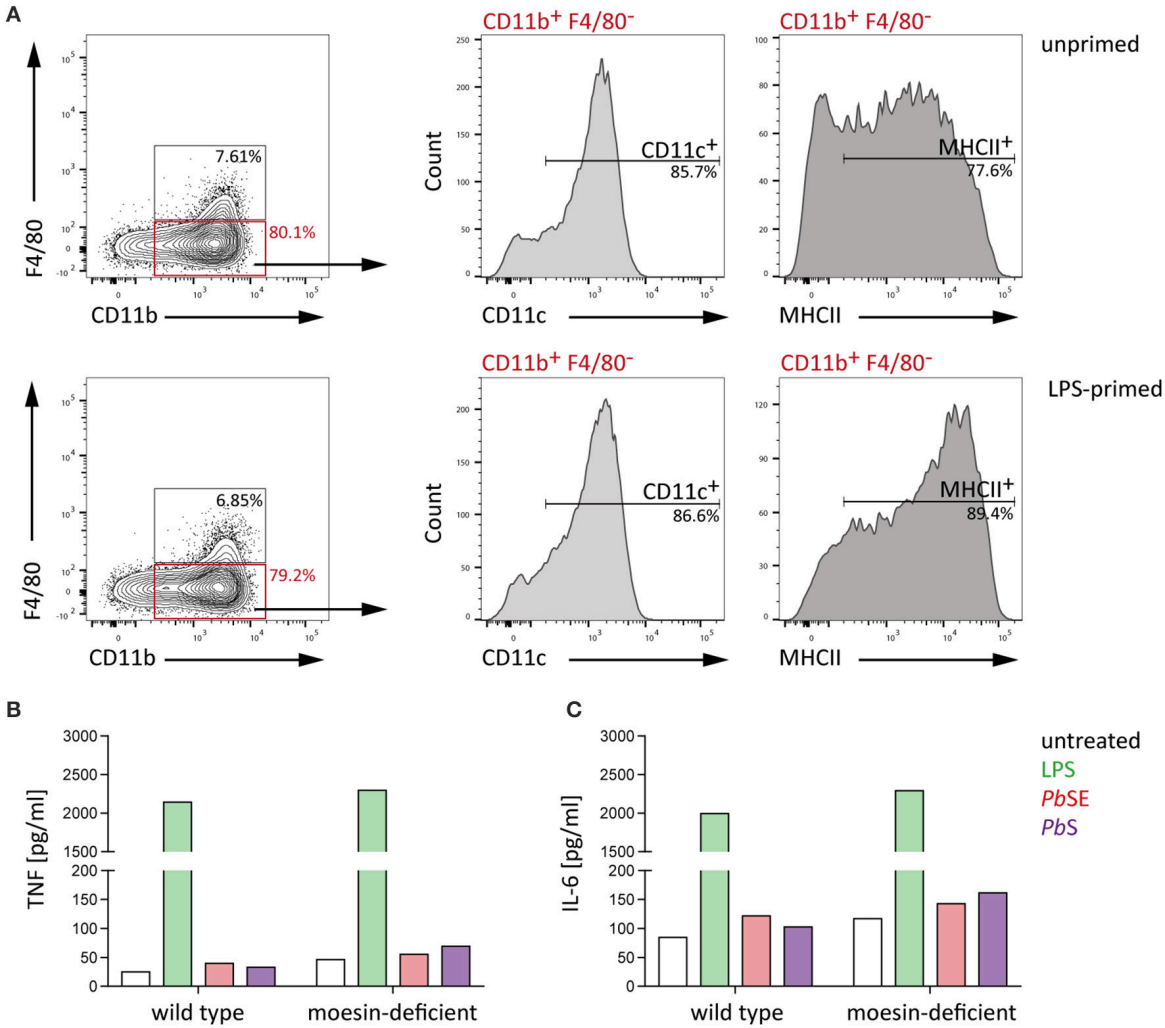
**WT and moesin-deficient BMDC respond similarly to stimulation**. BMDC were generated from bone marrow-derived cells of C57BL/6 WT mice using GM-CSF and IL-4 **(A)** Representative plots of flow cytometric analysis of BMDC differentiation with and without 24 h LPS priming; F4/80^+^ cells were excluded and CD11b^+^ cells analyzed for CD11c and MHCII surface expression, gates were set according to FMO controls and dead cells were excluded from analysis by using a fixable viability dye. **(B,C)** Response of unprimed WT and moesin-deficient BMDC to stimulation with LPS (10 ng/ml), *P. berghei* schizont extract (*Pb*SE, 1:1,000 dilution in medium), and *P. berghei* schizonts (*Pb* schizonts, added at a ratio of 1:10 to BMDC), determined after 24 h incubation by CBA; **(B)** TNF supernatant concentrations of stimulated BMDC, **(C)** IL-6 concentrations in supernatants of BMDC; Due to *n* = 1, no statistical analysis was performed.

In line with the observations of the present study, Wu et al. (2015) reported that macrophage responsiveness is impaired due to *Plasmodium*-induced phagosomal acidification and that early cytokine responses to *Plasmodium* infection are rather DC-mediated. Given that macrophages and DCs both recognize PAMPs via pattern recognition receptors and are capable of phagocytosis (Gazzinelli et al., 2014), the interaction of moesin and *Plasmodium* GPI may serve a similar function like that proposed for macrophages in DCs. Consequently, bone marrow-derived dendritic cells (BMDC) were generated and successful differentiation was confirmed by flow cytometric analysis of viable WT BMDC which expressed key DC surface markers, i.e., CD11b, CD11c, and MHCII (Lutz et al., 1999), while only few cells expressed the macrophage marker F4/80 (Figure 6A). In line with previous reports (Madaan et al., 2014), LPS-priming induced maturation of BMDC, as indicated by a population shift to high level MHCII expression (Villadangos et al., 2005), demonstrating that the cells are properly differentiated.

Next, WT and moesin-deficient BMDC were stimulated with LPS, *Pb*SE, and *P. berghei* schizonts and TNF as well as IL-6 concentrations in supernatants were analyzed by CBA. In good agreement with previous observations in BMDM, the LPS- and malaria PAMP-induced response of BMDC revealed subtle and inconsistent differences between WT and moesin-deficient BMDC for all conditions tested (Figures 6B,C). Even though the cytokine response to *Plasmodium*-derived stimuli is rather low, these results indicate that the BMDC response to these pathogen-derived stimuli as measured by TNF and IL-6 secretion is independent of the presence of moesin.

### Immune response to *P. berghei* ANKA and infection outcome are moesin-independent

**Figure 7:**
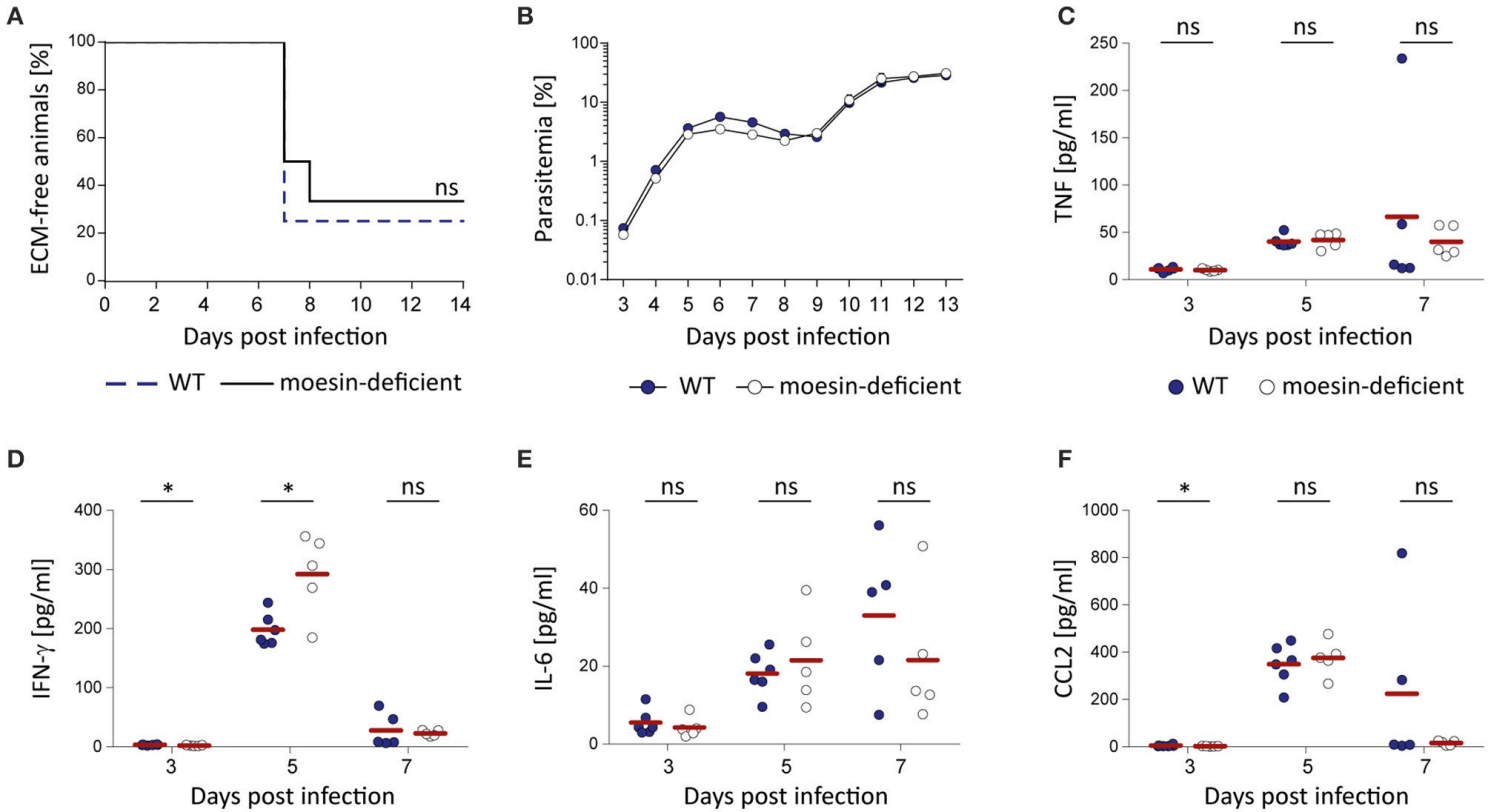
**Moesin-deficiency does not protect mice from ***P. berghei*** ANKA-induced ECM**. Moesin-deficient mice and corresponding WT controls were infected by i.v. injection of 10,000 *P. berghei* ANKA Bergreen-infected erythrocytes and the impact of moesin-deficiency on the course of infection and the systemic immune response was analyzed; **(A)** Survival curve indicating development of ECM in both WT and moesin-deficient mice, *n* = 12/genotype; Mantel Cox test revealed that differences are non-significant. **(B)** Representative parasitemia (*n* = 6/genotype) indicating similar progression of parasite growth independent of the host genotype. Parasitemia was determined by flow cytometric analysis of GFP^+^ erythrocytes; Dots display mean parasitemia + SD **(C–F)** Serum was collected from infected mice (*n* = 6 for WT, *n* = 5 for *Msn*^−/−^) at days 3, 5, and 7 post-infection by retrobulbar punction and analyzed for serum concentration of TNF **(C)**, IFN-γ **(D)**, IL-6 **(E)**, and CCL2 **(F)** by CBA. Dots represent individual mice, bars indicate means. Analysis by Mann-Whitney test: ^*^P < 0.05, ns, non-significant.

While a pivotal role of moesin-*Plasmodium* GPI interaction for macrophage responsiveness or functionality could not be demonstrated with the *in vitro* assays performed in this study, the interaction of moesin with *Plasmodium* GPI may be relevant in other *Plasmodium*-related host responses *in vivo*. Additionally, moesin may be involved in key events leading to the development of malaria pathology independent of its interaction with *Plasmodium* GPI, since moesin was described to be relevant in immunological synapse formation (Itoh et al., 2002; Parameswaran and Gupta, 2013) and in endothelial permeability (Koss et al., 2006; Yao and Tsirka, 2011). Thus, moesin-deficient C57BL/6 mice and the corresponding wild type controls were infected with *P. berghei* ANKA Bergreen blood stage parasites in order to investigate the potential involvement of moesin in the development of ECM. Despite the previously mentioned indications for moesin to play a role during processes leading to ECM, moesin-deficient mice were not protected from development of ECM (Figure 7A) concomitant with normal progression of parasite growth (Figure 7B). Moreover, lack of moesin did not affect the pro-inflammatory immune response mounted upon *P. berghei* ANKA infection in terms of serum levels of TNF, IL-6 and MCP-1/CCL2 (Figures 7C,E,F). Noteworthy, the interferon (IFN)-γ response differed significantly (*p* = 0.03) between WT and moesin-deficient mice at days 3 and 5 post-infection (Figure 7D), yet higher serum IFN-γ concentration at day 5 post-infection did not impact the overall course of infection in moesin-deficient mice.

## Discussion

In the present study, the ERM protein moesin was found to interact with *Plasmodium* GPI *in vitro* and the relevance of this interaction was further investigated in the context of *Plasmodium*-induced pro-inflammatory responses and pathology. Moesin is a protein of the ERM family, which undergoes conformational change upon phosphatidylinositol-4,5-bisphosphate (PIP_2_)-mediated phosphorylation (Neisch and Fehon, 2011) and links actin filaments to transmembrane proteins (Louvet-Vallee, 2000). Thereby, ERM proteins contribute to cytoskeletal rearrangement, cellular migration, and membrane dynamics (Ponuwei, 2016). In addition to ERM proteins interacting with phosphatidylinositide 3-kinase, protein kinase A, or Rho-specific GDP dissociation inhibitors (Ivetic and Ridley, 2004; Niggli and Rossy, 2008; Ponuwei, 2016), it has been suggested that LPS-induced TNF secretion is mediated by moesin signaling through the adapter protein MyD88 (Zawawi et al., 2010).

Moesin is a membrane-associated intracellular protein, which was previously reported to translocate to the cell surface upon LPS stimulation (Iontcheva et al., 2004; Takamatsu et al., 2009) and to be constitutively present on the surface of lymphocyte subsets (Takamatsu et al., 2009). Therefore, it was hypothesized that, upon translocation to the cell surface, moesin might interact with *Plasmodium* GPI. In contrast to previous reports on LPS-induced moesin cell surface translocation (Iontcheva et al., 2004; Takamatsu et al., 2009), we were unable to detect moesin on the cell surface of LPS-stimulated macrophage-like THP-1 cells or BMDM. Since intracellular moesin was detected in THP-1 cells and BMDM, and given that THP-1 cells as well as BMDM and BMDC responded to LPS-stimulation with TNF secretion, we were able to demonstrate that our protocol is generally suitable to detect moesin by flow cytometry and to elicit an LPS-induced pro-inflammatory cytokine response from different cell types. Although transient or nominal cell surface translocation of moesin upon LPS or *Pf* SE/*Pb*SE stimulation may not have been detected with the experimental settings used here, our results indicate permanent absence of moesin from the cell surface of macrophages *in vitro*.

The phagosome may be an alternative site for *Plasmodium* GPI and moesin to interact, since merozoite surface GPI become exposed upon schizont degradation. Moesin was described to be associated with phagosomes in murine J774 and human U937 macrophages (Desjardins et al., 1994; Defacque et al., 2000) and to be involved in phagosomal acidification (Erwig et al., 2006). However, in accordance with a previous study reporting that the rate of phagocytosis of apoptotic cells is moesin-independent (Erwig et al., 2006), moesin-deficiency did not affect non-opsonic uptake of *P. berghei* merozoites or schizonts in three independent experiments. Additionally, the absence of moesin did not have an impact on phagosomal degradation of *P. berghei* 18 s rRNA. Even though degradation of *P. berghei* 18 s rRNA was only analyzed once and at a limited number of time points, markedly reduced 18 s rRNA levels after 24 h of incubation in both wild type and moesin-deficient BMDM indicate that the presence of moesin is not critical for this process. Notably, the degradation of parasite components other than 18 s rRNA, e.g., hemozoin, was not quantified. Although Fc receptor-mediated phagocytic uptake of antibody-opsonized *P. berghei* merozoites or schizonts could be affected by moesin-deficiency, previously published data suggest that ERM proteins do not localize to phagosomes containing opsonized cells (Erwig et al., 2006).

Previous studies investigating the pro-inflammatory response to *Plasmodium* GPI demonstrated pronounced TNF secretion from BMDM *in vitro* using purified *Pf* GPI immobilized on gold particles (Krishnegowda et al., 2005; Zhu et al., 2009, 2011). Additionally, purified GPI of other protozoan parasites such as *Toxoplasma gondii* have been described to induce TNF secretion from murine macrophages *in vitro* (Debierre-Grockiego et al., 2003), indicating that protozoan GPI represent conserved PAMP. Although malaria PAMP other than GPI were present in the schizont extracts used here, which could activate pattern recognition receptors independent of moesin, we observed minor cytokine secretion and subtle induction of cytokine transcription in both wild type and moesin-deficient BMDM and BMDC. Thus, it seems unlikely that a potential contribution of moesin to *Plasmodium* GPI-induced signaling has been masked by activation of other pathways leading to cytokine production.

In line with our observation of a subtle induction of pro-inflammatory cytokines upon BMDM stimulation with *P. berghei* schizonts, another recent study reported that the cytokine response of BMDM is impaired upon internalization of *P. falciparum*- or *P. berghei*-infected erythrocytes or merozoites due to pronounced phagosomal acidification and consequential inactivation of endosomal TLR (Wu et al., 2015). Furthermore, the majority of splenic macrophages isolated from *P. berghei* ANKA-infected mice did not produce inflammatory cytokines (Wu et al., 2015). Collectively, our data support the notion that macrophages contribute marginally to the pro-inflammatory cytokine response at early stages of infection (Stevenson and Riley, 2004). Interestingly, it was recently demonstrated that *in vitro* stimulation of BMDC with *P. falciparum*-infected erythrocytes or merozoites resulted in cytokine secretion in a dose-dependent manner (Wu et al., 2015). In contrast, the BMDC cytokine responses to *Plasmodium*-derived stimuli observed here were subtle and inconsistent. Since the properties of BMDC generated *in vitro* vary depending on the stimuli used to induce differentiation (Xu et al., 2007), these BMDC may not properly reflect the spectrum of DC subtypes, e.g. monocyte-derived and plasmacytoid DC (Heath and Carbone, 2013), yet cytokine responses to *P. berghei* ANKA infection are DC subtype-dependent *in vivo* (Wu et al., 2015). However, cytokine secretion and transcription were markedly induced by LPS, suggesting that BMDC were generally proficient to respond to TLR ligands. Notably, despite previous reports on considerably reduced LPS-induced TNF secretion from THP-1 cells when moesin was silenced (Iontcheva et al., 2004) or blocked by anti-moesin antibody (Tohme et al., 1999; Amar et al., 2001; Zawawi et al., 2010), the LPS-induced TNF response was not affected by moesin-deficiency when compared to wild type BMDM or BMDC. Consequently, these results indicate that the LPS-induced cytokine response from murine BMDM and BMDC is moesin-independent *in vitro*. Although moesin likely serves similar functions in human and murine cells, further studies are needed to clarify the localization and the role of moesin in the pro-inflammatory immune response to LPS in human cells.

Moesin is the predominantly expressed ERM protein in T-cells (Itoh et al., 2002) and ERM proteins are involved in immunological synapse formation, thereby modulating T and B cell activation (Itoh et al., 2002; Parameswaran and Gupta, 2013). Furthermore, moesin is the predominant ERM protein in endothelial cells (Berryman et al., 1993) and was found to be involved in TNF-induced endothelial cell paracellular gap formation, resulting in increased endothelial permeability *in vitro* (Koss et al., 2006). Nevertheless, moesin-deficiency did not impair pro-inflammatory cytokine responses to *P. berghei* ANKA infection and did not affect ECM-associated mortality *in vivo*. These findings point toward a dispensable role for moesin as well as the interaction of moesin with *Plasmodium* GPI in the immune response to *P. berghei* ANKA infection and in the manifestation of symptoms of ECM.

Collectively, our findings demonstrate that even though *Plasmodium* GPI and moesin interact, the relevance of this interaction in the context of malaria pathology could not be established. Interestingly, although moesin has been described to be the predominantly expressed ERM protein in lymphocytes (Itoh et al., 2002) and endothelial cells (Berryman et al., 1993), it cannot be excluded that moesin-deficiency may be compensated for by other proteins of the ERM family, especially taking partial functional redundancy of ERM proteins into consideration (Niggli and Rossy, 2008). Additionally, given the conflicting *in vitro* data and considering that TLR-deficiency did not protect mice from ECM (Togbe et al., 2007; Lepenies et al., 2008), the precise mechanisms leading to the activation of the innate immune system during *Plasmodium* infection, as well as the relevance of GPI in this process, need to be further investigated to elucidate the underlying mechanisms of malaria pathology.

## Author contributions

FK conceived the study. JD, NA, XL, and FK designed and performed the experimental work. JD, NA, PS, and FK analyzed and interpreted data. ST provided moesin-deficient embryos. JD and FK wrote the manuscript. All authors approved the final version of the manuscript.

## Funding

This work was supported by the German Research Foundation grant to FK (KA 3347/4-1), the German Federal Ministry for Education and Research (BMBF) and the Max Planck Society.

### Conflict of interest statement

The authors declare that the research was conducted in the absence of any commercial or financial relationships that could be construed as a potential conflict of interest.

## Abbreviations

BMDC: bone-marrow derived dendritic cells
BMDM: bone-marrow derived macrophages
CM: cerebral malaria
DC: dendritic cells
ECM: experimental cerebral malaria
ERM: ezrin-radixin-moesin
FCS: fetal calf serum
FMO: fluorescence-minus-one
GPI: glycosylphosphatidylinositol
IFN: interferon
IL: interleukin
LPS: lipopolysaccharide
PAMP: pathogen-associated molecular pattern
*Pb*S: *Plasmodium* berghei schizonts
*Pb*SE: *Plasmodium* berghei schizont extract
*Pf* SE: *Plasmodium falciparum* schizont extract
PMA: phorbol 12-myristate 13-acetate
TLR: toll-like receptor
TNF: tumor necrosis factor
