# Supplementary material for: Interaction between Plasmodium Glycosylphosphatidylinositol and the Host Protein Moesin Has No Implication in Malaria Pathology
Source: Front Cell Infect Microbiol. 2017 May 16;7:183. doi: 10.3389/fcimb.2017.00183 (PMC5432536; doi:10.3389/fcimb.2017.00183)
Supplement: Supplementary file 1 [file Image1.PDF]

Supplementary Material

Interaction between *Plasmodium* glycosylphosphatidylinositol and the host protein moesin has no implication in malaria pathology

Josefine Dunst*, Nahid Azzouz, Xinyu Liu, Sachiko Tsukita, Peter H. Seeberger, and Faustin Kamena*

*** Correspondence:** Faustin Kamena: kamena@zedat.fu-berlin.de
and Josefine Dunst: dunstj@zedat.fu-berlin.de

Supplementary Figures

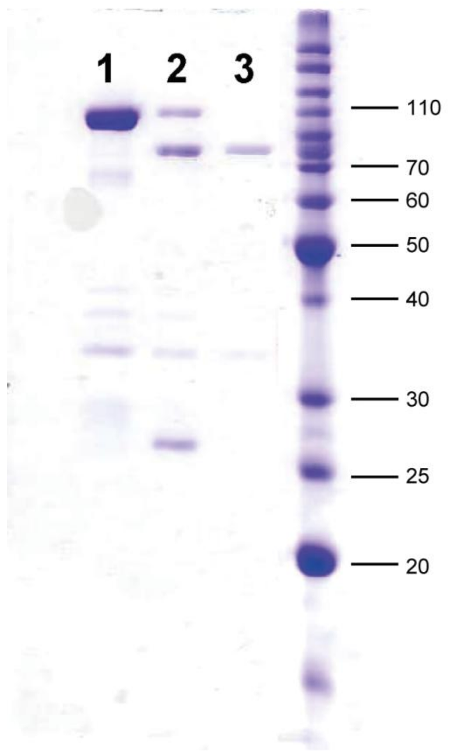

1- GST-Moesin

2- GST-Moesin after 2h cleavage with thrombin

3- Moesin after removal of cleaved GST

Supplementary Figure 1.

Thrombin cleavage of Moesin-GST: Purified Moesin-GST fusion protein (**1**) was incubated with immobilized thrombin Agarose for 2h at RT leading to the cleavage of the GST moiety (**2**). After centrifugation free moesin was recovered from the supernatant (**3**) and dialysed against PBS over night

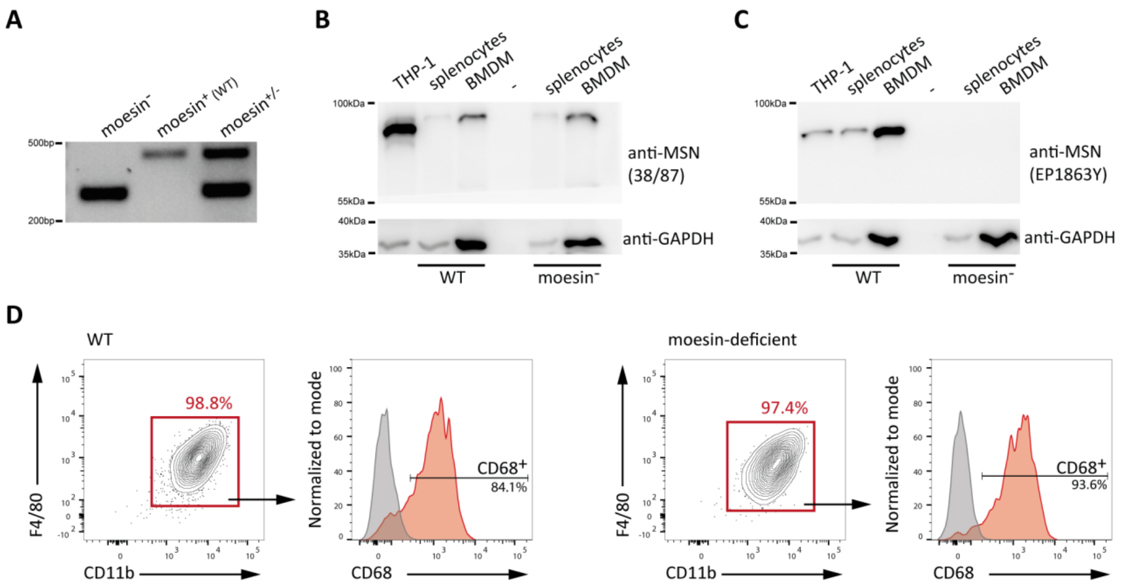

Supplementary Figure 2. Generation of wild type and moesin-deficient BMDM.

(**A**) Identification of moesin-deficient and WT mice by PCR using a combination of three primers; 300bp: moesin-deficient (moesin^{-}), 500bp: WT (moesin^{+}), two PCR products: heterozygous mice (moesin $^{+/-}$); (**B-C**) Western blot analysis of moesin protein expression by WT and moesin gene knockout mice: (B) Detection of moesin with anti-moesin antibody clone 38/87 suggests presence of moesin in all samples loaded (C) moesin detection with anti-moesin antibody clone EP1863Y indicates absence of moesin in moesin-deficient mice; THP-1 cells were used as positive control for moesin detection, GAPDH detection served as loading control. (**D**) Representative plots for flow cytometric analysis of viable BMDM for cell surface expression of CD11b and F4/80, as well as intracellular expression of CD68 in WT and moesin-deficient BMDM. Gating was performed with the respective fluorescence-minus-one (FMO) controls (grey histogram). Dead cells were excluded from the analysis using a fixable viability dye.
